# Airborne Transmission of SARS-CoV-2 Delta Variant within Tightly Monitored Isolation Facility, New Zealand (Aotearoa)

**DOI:** 10.3201/eid2803.212318

**Published:** 2022-03

**Authors:** Andrew Fox-Lewis, Felicity Williamson, Jay Harrower, Xiaoyun Ren, Gerard J.B. Sonder, Andrea McNeill, Joep de Ligt, Jemma L. Geoghegan

**Affiliations:** Counties Manukau District Health Board, Auckland, New Zealand (A. Fox-Lewis);; Auckland District Health Board, Auckland (F. Williamson, J. Harrower);; Institute of Environmental Science and Research, Porirua, New Zealand (X. Ren, G.J.B. Sonder, A. McNeill, J. de Ligt, J.L. Geoghegan);; University of Amsterdam, Amsterdam, the Netherlands (G.J.B. Sonder);; University of Otago, Dunedin, New Zealand (J.L. Geoghegan)

**Keywords:** COVID-19, airborne transmission, coronavirus disease, SARS-CoV-2, severe acute respiratory syndrome coronavirus 2, viruses, respiratory infections, zoonoses, New Zealand, Delta variant

## Abstract

In New Zealand, international arrivals are quarantined and undergo severe acute respiratory syndrome coronavirus 2 screening; those who test positive are transferred to a managed isolation facility (MIF). Solo traveler A and person E from a 5-person travel group (BCDEF) tested positive. After transfer to the MIF, person A and group BCDEF occupied rooms >2 meters apart across a corridor. Persons B, C, and D subsequently tested positive; viral sequences matched A and were distinct from E. The MIF was the only shared location of persons A and B, C, and D, and they had no direct contact. Security camera footage revealed 4 brief episodes of simultaneous door opening during person A’s infectious period. This public health investigation demonstrates transmission from A to B, C, and D while in the MIF, with airborne transmission the most plausible explanation. These findings are of global importance for coronavirus disease public health interventions and infection control practices.

Coronavirus disease (COVID-19) is a viral respiratory infection caused by severe acute respiratory syndrome coronavirus 2 (SARS-CoV-2) (*1*). Person-to-person transmission primarily occurs when respiratory particles containing SARS-CoV-2 are exhaled by an infected person and subsequently inhaled by others (*2*). Transmission through fomites is also possible but is considered to play a minimal role (*3*). Until recently, the principal route of COVID-19 transmission was thought to be through respiratory droplets (*4*; J.C. Palmer et al., unpub. data, https://www.medrxiv.org/content/10.1101/2021.10.19.21265208v1). Droplets are larger respiratory particles that fall quickly and thus disperse over short distances of generally <2 meters (6 ft) (*2*,*4*). However, evidence is emerging that the dominant route of COVID-19 transmission might in fact be airborne, through respiratory aerosols (*4*). Aerosols are smaller respiratory particles that remain suspended in the air for prolonged periods, and they can thus disperse and result in transmission over distances of >2 meters (*4*; J.C. Palmer et al., unpub. data). Epidemiologic studies are considered the most robust evidence currently available to support the biologic plausibility of airborne transmission of COVID-19 (*5*; J.C. Palmer et al., unpub. data).

To mitigate importation of COVID-19 into New Zealand (Aotearoa), border restrictions have been in place since March 2020; only citizens, permanent residents, and exempted persons have been permitted entry into the country (*6*). Persons entering the country must complete a period of quarantine in one of several government-assigned managed quarantine facilities (MQFs) that form part of the border response (*7*–*9*). While in a MQF, asymptomatic persons undergo mandatory SARS-CoV-2 screening tests by real-time reverse transcription PCR (rRT-PCR) of nasopharyngeal swab samples routinely collected on days 0, 3, and 12 after arrival in New Zealand, or as close to these times as practical (*10*). Symptomatic persons and MQF room companions of SARS-CoV-2–positive persons are tested as soon as possible after symptom onset or case identification (*7*,*10*). Persons who are identified as symptomatic at the border, have had a positive SARS-CoV-2 screening test, or who share the same MQF room as another SARS-CoV-2–positive person are immediately transferred from their respective MQF to a single dedicated managed isolation facility (MIF) for confirmed and suspected COVID-19 cases and close contacts.

Solo traveler A and a 5-person travel group, BCDEF, had traveled on different flights from different countries, arrived in New Zealand on different dates, and been staying in different MQFs. Persons A and E had positive SARS-CoV-2 screening tests, which resulted in the transfer of A and group BCDEF to the MIF, on different dates, where they occupied rooms across the corridor, 2.135 meters (7 ft) apart. Persons B, C, and D subsequently tested positive while in the MIF; viral sequences were linked by whole-genome sequencing (WGS) to person A, not to person E, who was within their travel group. A comprehensive epidemiologic investigation was undertaken by public health to determine whether airborne transmission of SARS-CoV-2 Delta variant had taken place between person A and persons B, C, and D, who were staying in separate, nonadjacent rooms >2 meters apart within the tightly monitored MIF.

## Methods

All nasopharyngeal swabs underwent routine rRT-PCR diagnostic testing by using the Cepheid Xpert Xpress SARS-CoV-2 assay (Cepheid, https://www.cepheid.com) or an E gene rRT-PCR laboratory-developed test on the Panther Fusion platform (Hologic, https://www.hologic.com) (*10*,*11*). WGS and phylogenetic analysis was undertaken as previously described (*12*,*13*). In brief, we assigned SARS-CoV-2 genomes from persons A, B, C, D, and E as lineage B.1.617.2 (Delta variant) by using Pangolin (*14*). We then aligned these genomes along with 1,000 Delta variant genomes uniformly sampled at random from GISAID (*15*) samples collected during July 1–14, 2021 using Nextalign (*16*), using the prototype strain Wuhan-Hu-1 (GenBank accession no. NC_045512) as reference. We estimated a maximum-likelihood phylogenetic tree by using IQ-TREE (*17*) using the best fit model and ultrafast bootstrapping for branch support.

COVID-19 is a notifiable disease in New Zealand: all PCR-confirmed cases are reported to Public Health for further investigation. Investigation outcomes of this in-facility transmission event have been communicated to the public by the New Zealand Ministry of Health (*18*,*19*). Because this was a public health investigation, formal ethics approval was not required (*20*). The 6 persons involved are anonymously described here as A–F, and because no identifiable details have been provided, formal written consent was not required. The infectious period was assumed to last up to 10 days after symptom onset or the first positive rRT-PCR test (*21*).

## Results

### Case Details

**Figure 1:**
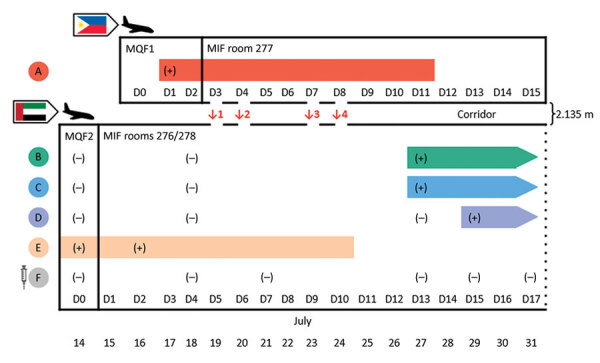
Timeline of infectious periods, test results, and relative locations of persons A–F, implicated in airborne transmission of severe acute respiratory syndrome coronavirus 2 (SARS-CoV-2) Delta variant between separate nonadjacent rooms within a tightly monitored MIF, New Zealand. Colors indicate persons A–F; bars represent each person’s infectious period of 10 days after symptom onset or the first positive rRT-PCR test. Syringe symbol indicates person was fully vaccinated against coronavirus disease. Person A occupied room 277 and travel group BCDEF occupied adjoining rooms 276 and 278 on the opposite side of the corridor in block 2 of the MIF. The doors to the rooms were 2.135 m apart. Map-arrow symbols indicate country of origin (Philippines and United Arab Emirates); airplane symbols denote date of arrival in New Zealand. Episodes of simultaneous door-opening between room 277 and rooms 276/278, each lasting 3–5 seconds, are indicated with ↓1 to ↓4. Positive SARS-CoV-2 rRT-PCR test results are indicated by (+); negative rRT-PCR test results are indicated by (–). MIF, managed isolation facility; MQF, managed quarantine facility.

**Figure 2:**
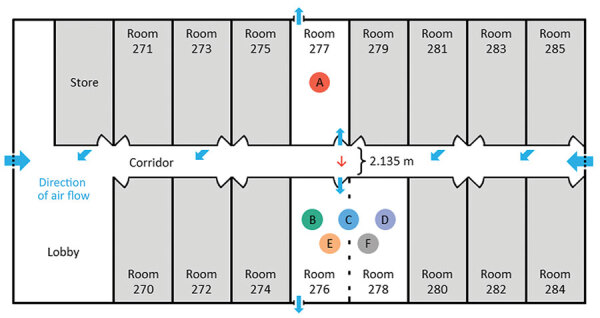
Layout of managed isolation facility block 2, New Zealand, in which airborne transmission of severe acute respiratory syndrome coronavirus 2 Delta variant occurred between separate nonadjacent rooms. Colored circles indicate persons A–F. Person A occupied room 277 and travel group BCDEF occupied adjoining rooms 276 and 278 on the opposite side of the corridor, 2.135 m apart. Red arrow indicates direction of probable airborne transmission of Delta variant from person A to persons B, C, and D. Blue arrows indicate direction of airflow.

Person A arrived in New Zealand from the Philippines on July 16, 2021, and was placed in MQF1. After a positive routine day 1 test result on July 17 (E gene cycle threshold [C_t_] value 20.57), person A was transferred to the MIF on July 19 (Figure 1) and placed in block 2, room 277 (Figure 2). Person A remained asymptomatic and had no further tests during the stay in the MIF. Person A was considered infectious up to and including July 27 and was released from the MIF on July 31.

Travel group BCDEF arrived in New Zealand from the United Arab Emirates on July 14 and were quarantined together in MQF2. One member of the group, person E, had a positive routine day 0 test result on July 14 (E/N2 gene C_t_ values 33.9/37.1). On July 15, the whole group was transferred to the MIF (Figure 1), where they were accommodated in block 2 in adjoining rooms 276 and 278 on the opposite side of the corridor from person A (Figure 2). The distance between the doors to room 277 and room 276 was 2.135 meters. Person E experienced upper respiratory tract infection symptoms on July 16–17 (coryza and subjective fever) and had a further positive SARS-CoV-2 rRT-PCR test on July 16 (E/N2 gene C_t_ values 15.6/17.3).

Person B experienced upper respiratory tract infection symptoms on July 17–18; on July 18, a rRT-PCR test result was negative for SARS-CoV-2 but positive for rhinovirus/enterovirus. Persons B, C, and D subsequently tested positive for SARS-CoV-2. Persons B and C had positive routine day 13 tests on July 27 (E/N2 gene C_t_ values 17.6/18.7 for person B and 17.2/18.9 for person C) but were not symptomatic. They had no further SARS-CoV-2 tests during their stay in the MIF. Person D had a negative day 13 test but had a headache on July 29 and tested positive for SARS-CoV-2 by rRT-PCR that day (E/N2 gene C_t_ values 25.3/27.3). Person D had a further positive test on August 9 (E/N2 gene C_t_ values 28.7/30.6).

Despite sharing a room with 4 other persons with PCR-confirmed SARS-CoV-2 infection, person F never tested positive for SARS-CoV-2 by rRT-PCR, testing negative on July 14, 18, 21, 27, 29, 31, and August 8, 14, 16, and 23. Person F had received 2 doses of the Pfizer-BioNTech (https://www.pfizer.com) COVID-19 vaccine, but no other members of the travel group had been vaccinated.

Travel group BCDEF remained together in the MIF until person F had completed the 14-day isolation period after the last SARS-CoV-2 exposure (14 days after August 8, the last day of the infectious period of person D). Travel group BCDEF were released from managed isolation on August 25.

### Viral Genomic Data

**Figure 3:**
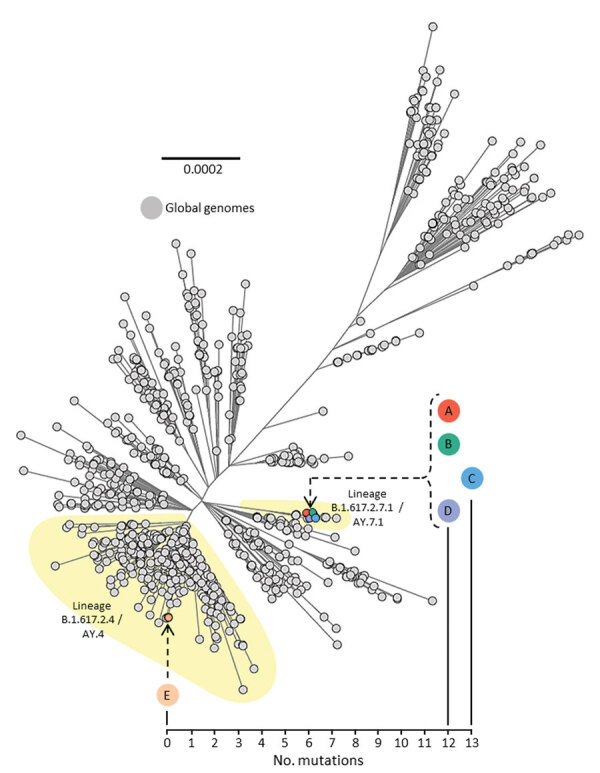
Unrooted maximum-likelihood phylogenetic tree of genomes from severe acute respiratory syndrome coronavirus 2 (SARS-CoV-2) isolated from persons A, B, C, D, and E, implicated in airborne transmission of SARS-CoV-2 Delta variant between separate nonadjacent rooms within a tightly monitored managed isolation facility, New Zealand, set among a background of other lineage B.1.617.2 (Delta variant) genomes sampled from around the world during July 1–14, 2021. Colored circles indicate persons A–E. Person F is not included because they were not infected with SARS-CoV-2 during the timeframe of this investigation. Upper left phylogenetic scale bar indicates number of nucleotide substitutions per site. Lower right scale shows number of mutations (single nucleotide polymorphisms) difference between viral sequences isolated from persons A, B, C, D, and E.

All samples testing positive for SARS-CoV-2 by rRT-PCR underwent WGS for routine surveillance purposes. Persons A, B, C, D, and E had all been infected with SARS-CoV-2 lineage B.1.617.2 (Delta variant). The viral genome sequence isolated from person A was SARS-CoV-2 sublineage B.1.617.2.7.1 (AY.7.1); the sequence was genetically identical to the sequences isolated from persons B and D and only 1 single-nucleotide polymorphism different from the sequence from person C (Figure 3). However, the viral genomes sequenced from these 4 persons (A, B, C, and D) were genetically distinct (difference of 12–13 single-nucleotide polymorphisms) from the SARS-CoV-2 Delta variant from person E, which was of a different sublineage, B.1.617.2.4 (AY.4). Genomic data for all 5 persons are available on GISAID (accession no. EPI_ISL_3164123 [person A], EPI_ISL_3477087 [person B], EPI_ISL_3477085 [person C], EPI_ISL_3477082 [person D], and EPI_ISL_3164111 [person E]).

### Exclusion of Laboratory Error

Initial investigative efforts focused on ruling out laboratory error to exclude a mix-up between samples from persons A and E. The sample from person A and the 2 samples from person E were collected on different dates from different locations, underwent diagnostic rRT-PCR testing at different laboratories, and were sequenced on separate runs at the national reference laboratory. Both samples from person E had already undergone WGS before collection of the positive samples from the subsequent cases in the same travel group (persons B, C, and D). The 2 samples from person E were genomically linked to each other; the July 14 sample (E/N2 gene C_t_ values 33.9/37.1) yielded a partial genome that matched the sample from July 16 (E/N2 gene C_t_ values 15.6/17.3), which was sequenced in full. These 2 samples from person E were genomically distinct from the positive samples from persons B, C, and D. These findings exclude a reversal of samples between persons A and E, refuting the hypothesis that person E was linked to subsequent cases in the travel group, rather than these cases being linked to person A, which we determined to be the case.

### Security Camera Footage

Review of closed-circuit television security camera footage from the MIF block 2 corridor during the period that person A was deemed to be infectious (July 19–27) revealed 4 separate episodes of simultaneous opening of doors to room 277 and room 276, each of which occurred for intervals of 3–5 seconds. In episode 1, on July 19, person A and a member of group BCDEF opened the respective doors for a food delivery at the same time (timeframe 4.2 s). In episode 2, on July 20, a member of group BCDEF opened the door for a food delivery and talked briefly to the delivering MIF staff member, then person A also opened the door for food, upon which the member of group BCDEF was instructed by the staff member to close that door (timeframe 3 s). In episode 3, on July 23, person A and a member of group BCDEF opened their respective doors for food delivery at the same time (timeframe 3–5 s). In episode 4, on July 24, a MIF nurse conducting a health check initially knocked on the door of room 277; after no answer, the nurse knocked on the door of room 276. However, the door to room 277 was opened first by person A, and then the door to room 276 was opened by a member of group BCDEF. The member of group BCDEF was told by the nurse to close that door while she undertook the health check on person A. After she completed the health check on person A, the door to room 277 was closed. The nurse cleaned equipment and changed gloves, then knocked on the door of room 276, which was opened by a member of group BCDEF (timeframe 4–5 s).

Person A was found to have not left the room at any point during their infectious period at the MIF and only left the room for exercise after the infectious period, from July 28 onward (after persons B and C had already tested positive). During the infectious period of person A, no fire evacuations or other drills at the MIF occurred that would have required guests to leave their rooms. Camera angles meant that security camera footage could not identify which member of group BCDEF opened the doors in the episodes previously described. In addition, security camera footage could not confirm that medical masks were worn by the persons answering the doors, but wearing of medical masks when opening doors is standard policy in the MIF.

The MIF delivery staff involved in the simultaneous door-opening episodes 1–3 wore medical masks and gloves during these encounters and were positioned >2 meters away from the rooms when the doors were open. The nurse involved in simultaneous door-opening episode 4 was wearing full personal protective equipment, including gloves, gown, goggles, and an N95 particulate respirator. The staff identified as being involved in these interactions had all received 2 Pfizer-BioNTech COVID-19 vaccinations and underwent weekly surveillance rRT-PCR testing; each staff member had >3 negative test results after these encounters. No other persons within the facilities had SARS-CoV-2 genomes linked to these cases.

### Room and Corridor Air Ventilation

**Figure 4:**
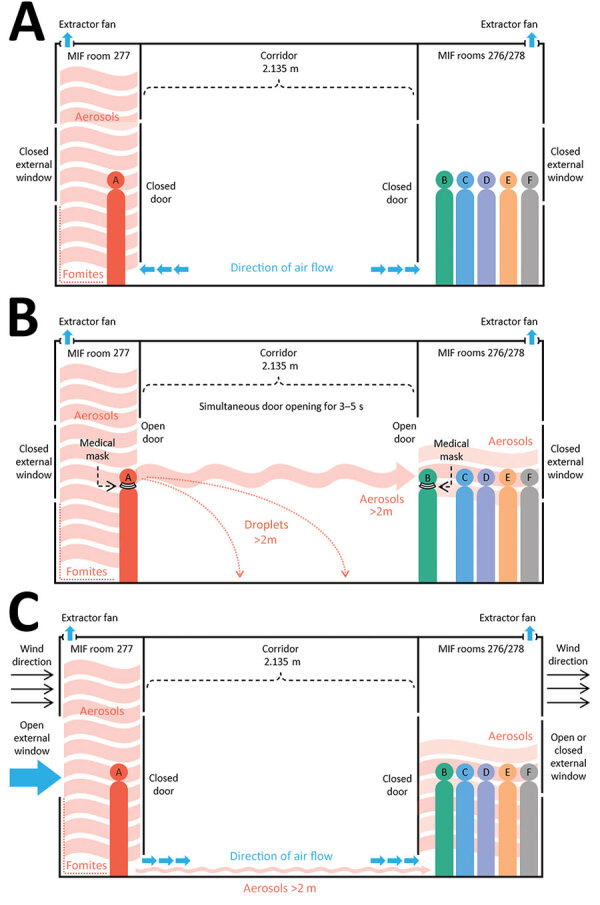
Possible mechanisms of airborne transmission of severe acute respiratory syndrome coronavirus 2 (SARS-CoV-2) Delta variant between separate nonadjacent rooms within a tightly monitored MIF, New Zealand. A) Air flow through rooms when room doors and external windows are closed; rooms are negative pressure and air moves from the corridor into the rooms, exiting by extractor fans. B) Movement of viral aerosols between rooms during episodes of simultaneous door-opening, when negative pressure generated by extractor fans is negated. C) Movement of viral aerosols under room doors, aided by opening of external room windows and outdoor meteorological conditions (wind speed and direction), which can create internal air flows within the building. Colored circles indicate persons A–F. Blue arrows indicate direction of air flow. Different types of infectious particles are annotated in red, with all infectious particles originating from person A. Red arrows indicate direction of movement of infectious particles. Person B is shown opening the door in this example; however, security camera footage could not identify which group member opened the door during the episodes. Security camera footage could not confirm that masks were worn by the persons answering the doors, but wearing of medical masks when opening doors is mandated in the MIF. MIF, managed isolation facility.

Before this investigation, the negative pressure capabilities of the MIF rooms had been assessed. Within the ensuite bathroom of each room was a continuously operating extractor fan, with an average extraction rate of 36 L/s (128 m^3^/h). The extractor fan removed air from the room, venting it to the outside and generating an average negative pressure of approximately −6.6 Pa in each room. The US Centers for Disease Control and Prevention engineering specifications for negative pressure rooms recommend a negative pressure exceeding −2.5 Pa (*22*). Smoke tests performed in a sample of rooms had confirmed a gradual but definite observed flow toward the closed bathroom door. Each room was equipped with a free-standing high efficiency particulate air (HEPA) filter, which recirculated and filtered air within the room but did not affect air movement into or out of the room. No ventilation systems connected separate rooms. Rooms had external windows that could be freely opened by occupants. External air was pumped into the corridor at either end, which, coupled with the room extractor fans, meant that when room doors and external windows were closed, the direction of air flow was from the corridor into the rooms (Figure 4, panel A).

A total of 4 free-standing HEPA filtration units were present in the MIF block 2 corridor (Figure 2). Investigation of the outward air flow from these units revealed that air exited the units in the horizontal plane at an angle of ≈45 degrees from the wall. The nearest unit to rooms 277 and 276 was mounted on the wall outside room 281, on the same side of the corridor as room 277. Air flowed out of this unit diagonally from one side of the corridor to the other (i.e., from the door of room 277 to the door of room 276).

## Discussion

We concluded that an episode of airborne transmission of SARS-CoV-2 Delta variant occurred between person A, the index case-patient, and persons B, C, and D, the secondary case-patients, who were staying in separate nonadjacent rooms 2.135 meters apart within the MIF. This conclusion is supported by multiple lines of evidence.

First, transmission between person A and persons B, C, and D could only have occurred within the MIF. This facility was the only location where these persons were colocated, because person A and travel group BCDEF had traveled on different flights from different countries, arrived in New Zealand on different dates, stayed in different MQFs, and were transferred to the MIF on different dates. Second, person A and travel group BCDEF were located in relatively close physical proximity within the MIF, in rooms across the corridor from one another. Third, the infectious period of person A preceded infection in persons B, C, and D. Fourth, during the infectious period of person A, several episodes of simultaneous door-opening occurred between the rooms occupied by person A and travel group BCDEF, meaning that for a short time no barriers to the spread of airborne respiratory aerosols between these rooms were in place. Fifth, during the episodes of simultaneous door-opening, person A and the member of travel group BCDEF who opened the door should have been wearing medical masks, as is mandated within the MIF. The wearing of medical masks, short duration of simultaneous door-opening, and separation by >2 meters makes transmission by droplets improbable. Sixth, the risk for fomite transmission of SARS-CoV-2 by shared surfaces is already thought to be low (*3*). Person A and travel group BCDEF had no direct contact with each other or with any shared objects, as corroborated by security camera footage, making transmission by fomites in this case also improbable. Finally, viral genomic data demonstrate that persons A, B, C, and D had genetically identical or closely linked SARS-CoV-2 Delta variant viral genomes and that these were markedly different from the Delta variant genome sequenced from person E. The cumulative evidence of these findings indicates that transmission of SARS-CoV-2 Delta variant took place between person A and persons B, C, and D during their stay in the MIF and that transmission by an airborne route is the most plausible explanation.

Like many such facilities globally, the MIF described here was not built for this function but rather was a commercial hotel complex that had been adapted for use as a MIF in the wake of the COVID-19 pandemic (*9*). Although the rooms did have negative pressure capabilities, they did not have anterooms to maintain negative pressure during entry and exit, and they had external windows that could be freely opened by occupants. Opening either the door to the corridor or the external window could negate the negative pressure within the room, enabling aerosol particles to disperse out of rooms. Person A did not leave the room at any point during their infectious period, likely resulting in a high concentration of viral aerosols accumulating in the room. Our findings support the hypothesis that during episodes of simultaneous door-opening, airborne particles in the room of person A rapidly diffused down a concentration gradient, across the corridor, and into the rooms of group BCDEF (Figure 4, panel B). Air flow from the corridor HEPA filter outside room 281 could have aided in aerosol movement across the corridor (Figure 2). This explanation is more plausible than exhalation and transmigration of viral aerosols only during the brief periods of simultaneous door-opening.

Another potential mechanism for movement of viral aerosols between opposite rooms is air flow under the room doors (Figure 4, panel C). As previously described, continuously operating extractor fans generate negative pressure in the rooms, causing air to flow from the corridor, under closed room doors, and into the rooms. Opening external room windows could negate the negative pressure generated by the extractor fans and permit external weather conditions to influence internal air flow within the building (*23*). Transmission of SARS-CoV-2 during brief periods of simultaneous door-opening or because of subtle internal air flows under room doors highlights the highly infectious nature of the Delta variant, especially in indoor settings. Transmission by an intermediary case, such as a MIF staff member, is highly unlikely given that all MIF staff members are fully vaccinated against COVID-19 and have weekly surveillance SARS-CoV-2 rRT-PCR testing and that no staff members tested positive for SARS-CoV-2 in the weeks surrounding this event.

Locally, the outcome of this investigation effected an immediate change in food delivery and health check protocols at the MIF to eliminate episodes of synchronous door opening. Corridor HEPA filtration units were reoriented so that air exited the units parallel to the wall to mitigate against movement of respiratory aerosols across the corridor. In addition, depending on occupancy, future room allocation of residents within the MIF will be spread out as much as possible.

Genomic epidemiologic studies such as this one provide the best evidence currently available to support airborne transmission of SARS-CoV-2, the causative agent of COVID-19 (J.C. Palmer et al., unpub. data). The findings of this comprehensive public health investigation describing airborne transmission of SARS-CoV-2 are vital for global public health interventions and infection prevention and control practices relating to COVID-19. The findings are relevant to healthcare settings, managed quarantine and isolation facilities, and other community indoor environments. To date, multiple reports of SARS-CoV-2 transmission over distances incompatible with droplet spread exist (J.C. Palmer et al., unpub. data), including epidemiologic studies from isolation hotels such as the one we describe in this study (*9*,*24*). This study adds key information to the growing body of evidence supporting a primarily airborne route of transmission for COVID-19 (*4*).
